# Supplementary material for: Perceptions towards the COVID-19 Pandemic during Different Lockdown Levels among International Students in Taiwan
Source: Int J Environ Res Public Health. 2023 Mar 11;20(6):4944. doi: 10.3390/ijerph20064944 (PMC10049484; doi:10.3390/ijerph20064944)
Supplement: Supplementary file 1 [file ijerph-20-04944-s001.zip › ijerph-2235927-supplementary.pdf]

Supplementary Material File S1

Questions for knowledge, attitudes, practices, impacts and information source

Knowledge

1. People infected with SARS-CoV-2 (virus causing COVID-19) cannot transmit the virus to others if fever is not present.
2. SARS-CoV-2 (virus causing COVID-19) spreads through respiratory droplets only when infected people cough and sneeze.
3. COVID-19 can be contracted by touching one's mouth, nose or perhaps, eyes after contacting a surface or object, on which the virus is attached.
4. The general population can wear medical (surgical) masks to help prevent the COVID-19.
5. Isolation of people with COVID-19 is an effective way to reduce the spread of virus.
6. People in contact with someone with COVID-19 should be immediately quarantined and observed, in an appropriate location, for at least 14 days.
7. Elderly and those with underlying health conditions are more likely to have severe diseases or die of COVID-19.

Attitudes

1. It is important to keep social distance from others, in order to protect myself from COVID-19.
2. Washing hands is essential to protect myself from COVID-19.
3. It is important to wear surgical mask to protect myself from COVID-19.
4. My school provided sufficient information of COVID-19 for me.
5. The preventative measures adopted by my school are effective to decrease the spread of COVID-19.
6. The government of Taiwan provided sufficient information of COVID-19 for me.
7. Compliance with the Taiwan governmental regulations is effective to prevent the spread of COVID-19.
8. Vaccination is the ultimate method to control COVID-19.

Practices

1. During the epidemic, have you been practicing social distancing?
2. Do you use soap when washing your hands?
3. Do you avoid cultural behaviors, such as shaking hands, kissing or hugging during the epidemic?
4. Do you carry hand sanitizer with you during the epidemic?
5. Do you wear a mask in public places to protect yourself against COVID-19?
6. During the epidemic, do you avoid visiting clinics / hospitals?
7. Do you follow your school's regulations for COVID-19?
8. Do you follow the Taiwan governmental regulations for COVID-19?

Impacts

1. Do you change your behavior in the following area?
   A. Transportation, commute
   B. Study
   C. Leisure
   D. Family life (including child bearing)
   E. Diet behavior
   F. Income

Information source

1. Which source(s) of information you access the most to learn about COVID-19?
   A. Printed media (Newspaper)
   B. Social media (Twitter, Facebook, WhatsApp, Line, Instagram)
   C. Government channels (Website/Local television/Electronic news)
   D. Friends/Neighbors/Relatives
   E. Others
2. Which of the following information source is most reliable?
   A. Friends/Classmates/Schoolmates
   B. Research results & scientific journals
   C. Taiwan government
   D. World Health Organization
   E. Information released by your university
   F. Others
